# Examining Methylation at the Intersection of Genetic Risk To Alzheimer’s Disease And Alcohol Use Disorder

**DOI:** 10.1002/alz.086508

**Published:** 2025-01-03

**Authors:** Gita Pathak, Robert H Pietrzak, Daniel Levey, Janitza Montalvo‐Ortiz, Henry Kranzler, Joel Gelernter, Renato A Polimanti

**Affiliations:** ^1^ Yale School of Medicine, West Haven, CT USA; ^2^ Yale University, New Haven, CT USA; ^3^ 3Center for Studies of Addiction, University of Pennsylvania Perelman School of Medicine and Crescenz VAMC, Philadelphia, PA USA; ^4^ Yale University School of Medicine, New Haven, CT USA

## Abstract

**Background:**

Alcohol Use Disorder (AUD) affects over 15 million individuals in the United States, contributing to oxidative stress, neuroinflammation, and elevating the risk of neurodegeneration. Despite this, the connection between AUD and aging conditions, particularly Alzheimer’s disease (AD), remains unclear. AD, with a heritability of 60‐80%, is genetically linked, necessitating an exploration of the molecular implications of AUD and genetic susceptibility to AD.

**Method:**

Participants were assessed for AUD using DSM‐5 criteria, and genotype data were imputed with the TOPMED reference panel. The polygenic score (PGS) of AD was calculated in European ancestry participants (mean±SD, 40.12 ±13.3 yrs; N = 4,915), adjusting for age, sex, and genetic ancestry. Methylation was assayed using the Illumina EPIC array using blood to conduct an epigenome‐wide association study (EWAS) on the interaction between PGS‐AD and AUD at 657,226 CpG sites in a subset of participants (N = 452). Intrinsic and extrinsic epigenetic age acceleration (IEAA, EEAA) were calculated using Hannum and Horvath’s epigenetic clocks.

**Result:**

We observed a significant association between PGS of AD and AUD diagnosis (cases = 34%; OR = 1.44; p = 0.005). The subsequent EWAS revealed top CpG sites in genes associated with AD pathogenesis, including *RLF, CD2AP, GLUL, GJA3, TRIM26, JAG2, RERE, SRRM3, COL11A2*. For these top CpGs, we investigated the beta (methylation intensity) in individuals with high‐PGS of AD (top 25% quantile) and stratified them by AUD diagnosis. Briefly, the top two CpG sites in AUD cases‐cg01101221(*RLF*; p = 5.51E‐07) was hypomethylated (p = 0.0205); and cg05804888(*CD2AP*; p = 4.61E‐06) was hypermethylated (p = 0.0142). We also observed interaction of AUD and PGS of AD is associated with accelerated aging‐ IEAA and EEAA from both Horvath and Hannum’s clocks (p<8.56E‐06<0.0092).

**Conclusion:**

The identified genes have been implicated in AD pathogenesis, such as *CD2AP’s* association with Aβ production and *GLUL*’s role in brain lipid metabolism. The study also highlighted the association between AUD and PGS‐AD interaction and accelerated epigenetic aging. While the CpG associations did not reach Bonferroni significance, our ongoing research aims to extend these findings to other ancestries and cohorts to improve statistical power. Thus providing a comprehensive understanding of the complex interactions between AUD diagnosis and genetic susceptibility to AD.

